# Combination Therapy of 5-Fluorouracil and Triamcinolone Acetonide with Compression Therapy after Surgical Excision in the Management of Keloids in Ears

**DOI:** 10.1055/s-0044-1801403

**Published:** 2025-01-17

**Authors:** Randeep Singh Lamba, Pinki Pargal, Anurag Salwan, Junaise P.M, Pallavi Nigam, Noopur Bansal

**Affiliations:** 1 Department of Plastic and Reconstructive Surgery, Christian Medical College, Ludhiana, Punjab, India

**Keywords:** 5-fluorouracil, compression therapy, keloid, triamcinolone acetonide

## Abstract

**Background** Any deviation from the normal process of wound healing leads to excessive scar formation in the form of keloid or hypertrophic scar.

**Materials and Methods** The study included 120 candidates with keloids divided equally into two groups, A and B, of 60 patients each. After surgical excision, group A received combination therapy of intramarginal 5-fluorouracil (5-FU) and triamcinolone acetonide (TCA), while group B received only TCA, followed by compression therapy in both.

**Results** Eighty-seven patients had keloids on ear lobules, 25 (20.8%) on helix, and 8 (6.7%) over multiple locations on ear. Ninety-two (76.7%) had keloids over bilateral, 18 (15%) on left, and 10 (8.3%) on right ear. Sixty-three (52.5%) belonged to third, 65 (54.2%) to fourth, and 8 (6.7%) to fifth decade of life. Overall recurrence rate was 21.7 and 38.3% in group A and B, respectively. Recurrence was seen in 2 from group A (male:female 2:0) and 4 from group B (male:female 3:1) at 3 months, in 7 from group A (male:female 5:2) and 13 in group B (male:female 8:5) at 6 months, and in 4 from group A (male:female 3:1) and 6 from group B (male:female 5:1) at 1 year. Overall, pain was reported by 9 and 7 from group A and B, respectively, and burning sensation by 3 and 1 from group A and B, respectively. Ulceration was noted in 2, wound dehiscence in 1 and transient hyperpigmentation in 2 from group A. Based on the Vancouver Scar Scale, outcome on follow-up had an average of 3.5 at 3 months, 4.2 at 6 months, and 4.8 at 1 year in group A, and 3.8 at 3 months, 4.7 at 6 months, and 5.4 at 1 year in group B.

**Conclusion** Multimodal approach of combination therapy of intramarginal 5-FU and TCA with compression therapy after surgical excision of keloids in ears yields lower recurrence rate when compared with TCA alone. Chances of recurrence are more common in males than females. Though intramarginal 5-FU in combination with TCA has more localized side effects than TCA alone yet lower recurrence rate and better results in the long term can overcome the mild severity of these side effects.

## Introduction

The word “keloid” is derived from Greek word cheloide, meaning “crab” claw-like appearance. 1

Keloids are excessive scars extending beyond the margins of surgical insults or wounds differentiating it from hypertrophic scars.

The management of keloids varies from intralesional corticosteroids, 5-fluorouracil (5-FU), bleomycin, interferon α-2b, verapamil, imiquimod, pressure therapy, silicone products, radiotherapy, lasers, to surgical excision. 2 Management of keloids is debatable owing to its pathophysiology and high chances of recurrences increasing the plight of the patient, and being cumbersome for the surgeon.

5-FU is classified as an antineoplastic agent that inhibits deoxyribonucleic acid and ribonucleic acid synthesis. It is a pyrimidine analog used commonly for its antimetabolite and chemotherapeutic properties. It induces fibroblast apoptosis without necrosis. 5-FU inhibits the expression of the type I collagen gene in keloid fibroblasts. The side effects of 5-FU therapy include pain, burning, hyperpigmentation, and ulceration at the injection site. Skin erythema and ulceration are common adverse effects with 5-FU.

Intralesional corticosteroids, namely, triamcinolone acetonide (TCA) has been the most commonly used agent to treat keloids. The side effects of 5-FU can be overcome by injecting a combination of 5-FU and TCA. 3

TCA has been the most commonly used intralesional drug in the management of keloids with regression being noted in 50 to 100% cases, with a recurrence rate of about 33% after 1 year and 50% after 5 years when used as a monotherapy drug. 4

Pressure therapy of at least 24 mm of Hg can play a role by releasing matrix metalloproteinase-9 and prostaglandin E2, leading to fibroblast death because of tissue hypoxia and eventually leading to remodeling of the scar. 5 6 7 8

The ideal treatment modality for keloids is still under evaluation. The aim of this study was to assess and achieve recurrence-free outcome with minimal side effects in patients with keloids in ears by use of a multimodal approach in the management of keloids of ears with intramarginal TCA with 5-FU and compression therapy after surgical excision, and minimize the chances of recurrence.

## Materials and Methods

After necessary ethical clearance for this study, all the patients visiting the outpatient department of the department of plastic and reconstructive surgery with clinically diagnosed keloids in ears from March 2022 onwards were included in this study. This amounted to 120 patients with clinically diagnosed keloids in ears over a span of 2 years from March 2022 to March 2024, who were included in this study during which each patient was evaluated for 1 year. Informed consent was duly obtained from all individual participants included in the study in their vernacular language, which included their consent for exercising their free power of choice and to be included in this study. They were satisfactorily informed by the attending doctor, in a manner and language that they understand, the purpose of the study, the nature of investigations, and the procedures, along with awareness of their right to opt out of the trial at any time during the course of the study without having to give reasons for doing so. They had been told about the details of the procedures of the study and opted to take part in the investigations as required.

The inclusion criteria were patients between 18 and 50 years, keloids present in ears, and size of keloids less than 5 cm.

Exclusion criteria were pregnant females, lactating mothers, keloids more than 5 cm in size, patients with other skin diseases, patients with history of chronic renal and/or liver disease(s), patients allergic to xylocaine, TCA, and/or 5-FU, patients not willing to be included in the study, and patients lost on follow-up.

The patients were randomly divided in two groups by sealed envelope method. Patients in group A ( *n* = 60) underwent management with combination of intramarginal 5-FU and TCA and group B ( *n* = 60) underwent management with intramarginal TCA only, after complete surgical excision followed by compression therapy in both the groups.

All the procedures were performed as a day case surgery and all the patients were discharged on the same day after the surgical intervention.

After taking an informed consent, and under all aseptic precautions, 2% xylocaine was injected around the keloids. Elliptical incisions were made, and complete surgical excision of keloids was done till the junction of the normal surrounding tissue. Wound closure was done with 4-0/5-0 nonabsorbable nylon sutures after achieving hemostasis. Aseptic dressings were done with sterile surgical strips. Sutures were removed on 5th to 7th day postoperatively depending upon the wound status.

First dose of intramarginal injection of a combination of TCA and 5-FU or TCA alone was given on the 15th postoperative day in group A and B, respectively.

In group A, 1 mL single-use insulin syringe was used with combination of 0.2 to 0.5 mL/cm ^2^ of TCA (40 mg/mL) along with 0.2 to 0.5 mL/cm ^2^ of 5-FU (50 mg/mL) and injected intramarginally till blanching was seen.

In group B, similar 1 mL insulin syringe was used to infiltrate 0.2 to 0.5 mL/cm ^2^ TCA (40 mg/mL) alone intramarginally till blanching was seen.

Second dose of similar intramarginal drug(s) was given at 1 month postoperatively.

Third dose of similar intramarginal drug(s) was given at 2 months postoperatively.

**Fig. 1:**
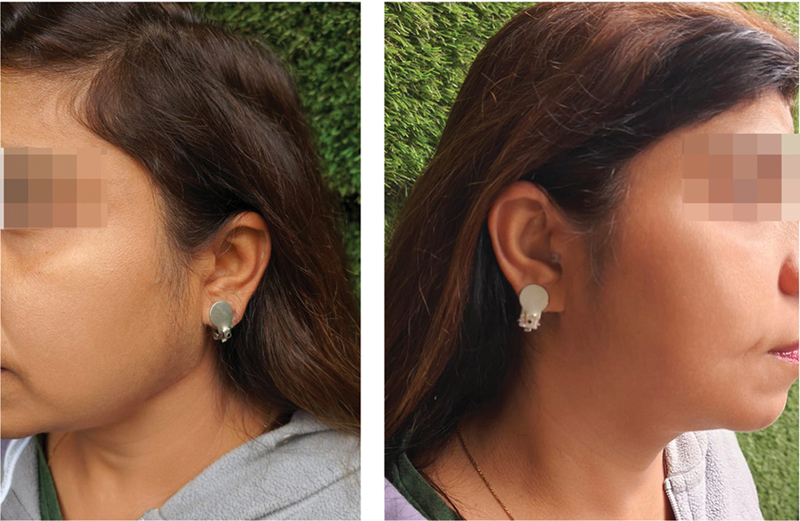
Customized metallic clips with silicone lining for compression therapy.

For compression therapy, application of customized metallic clips with approximately 0.5- to 1-mm thick silicone lining was done 1 week after suture removal for 3 months ( Fig. 1 ).

Patients were reviewed for a span of 1 year at 3 months, 6 months, and 1 year postoperatively.

Scar outcomes were evaluated on the basis on the Vancouver Scar Scale (VSS). 9

Note that 0 to 3 was rated as excellent, 4 to 6 as good, 7 to 10 as bad, and 11 to 13 as poor outcome.

## Results

Eighty-seven (72.5%) patients had keloids on ear lobules only, 25 (20.8%) patients had keloids on helix of the ear only, and 8 (6.7%) patients had keloids over multiple locations on the ear.

Ninety-two (76.7%) patients had keloids over bilateral ears, 28 (23.3%) had keloids on either of the two ears, 18 (15%) on the left ear, and 10 (8.3%) patients on the right ear.

Group A had 39 females and 21 males. Group B had 37 females and 23 males.

Patients from 18 to 50 years were included in this study. Sixty-three (52.5%) patients in this study belonged to the third decade of life, 65 (54.2%) to the fourth, and 8 (6.7%) to the fifth decade of life.

Ninety-eight (81.7%) patients had no history of any previous treatment for keloids, whether surgical, medical, or both.

**Fig. 2:**
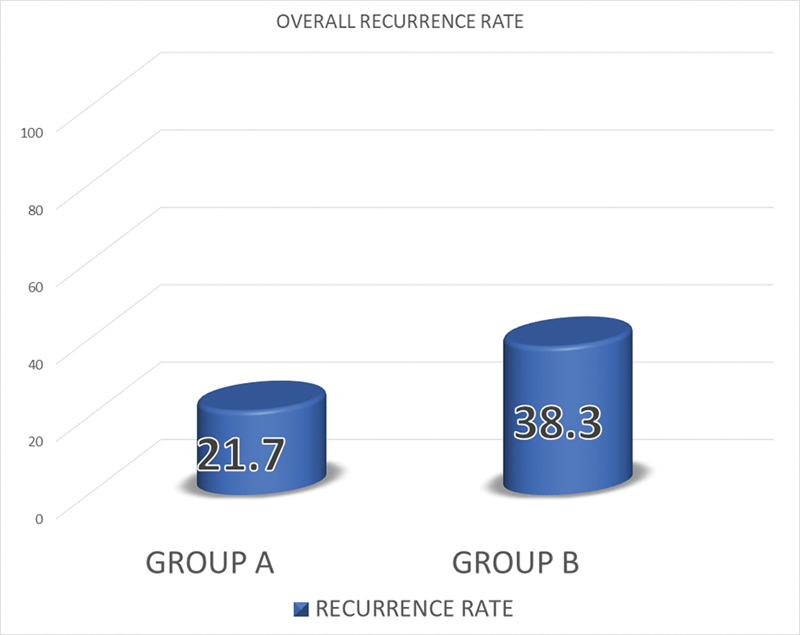
Overall recurrence rate.

Overall, the recurrence rate was 21.7% (13) in group A as compared with 38.3% (23) in group B, which had a *p* -value of 0.0214, which is statistically significant ( Fig. 2 ).

Overall, the recurrence rate in Group A was 47.6% (10) in males and 7.7% (3) in females, as compared to 38.3% (23) in males and 18.9% (7) in females in group B.

At 3 months, recurrence was seen in 2 patients in group A (male:female 2:0) and 4 patients in group B (male:female 3:1).

At 6 months, recurrence was seen in 7 patients in group A (male:female 5:2) and 13 patients in group B (male:female 8:5).

At 1 year, recurrence was seen in 4 patients in group A (male:female 3:1) and 6 patients in group B (male:female 5:1) ( Figs. 4 5 ).

**Fig. 3:**
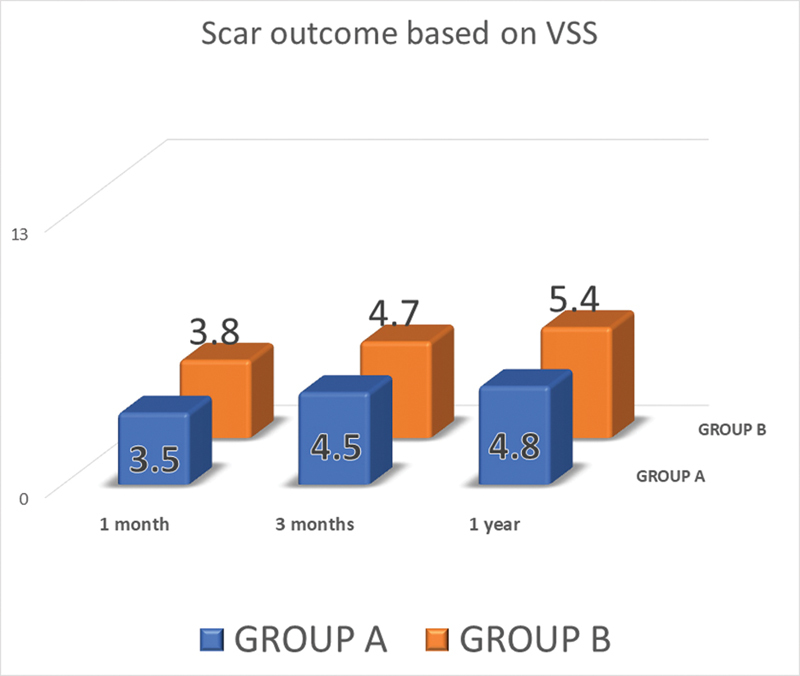
Scar outcome based on the Vancouver Scar Scale (VSS).

**Fig. 4:**
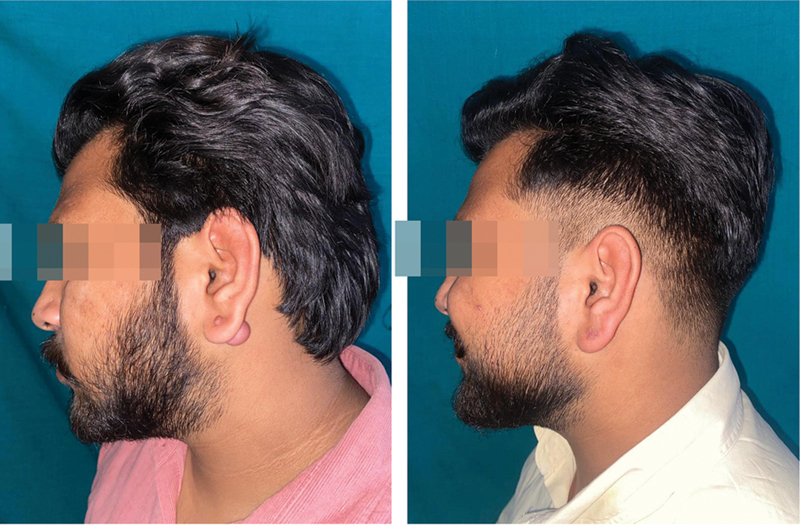
Patient A outcome before and after 1 year following surgical excision of left ear keloid followed by intramarginal combination therapy of 5-fluorouracil (5-FU) and triamcinolone acetonide (TCA) along with compression therapy.

**Fig. 5:**
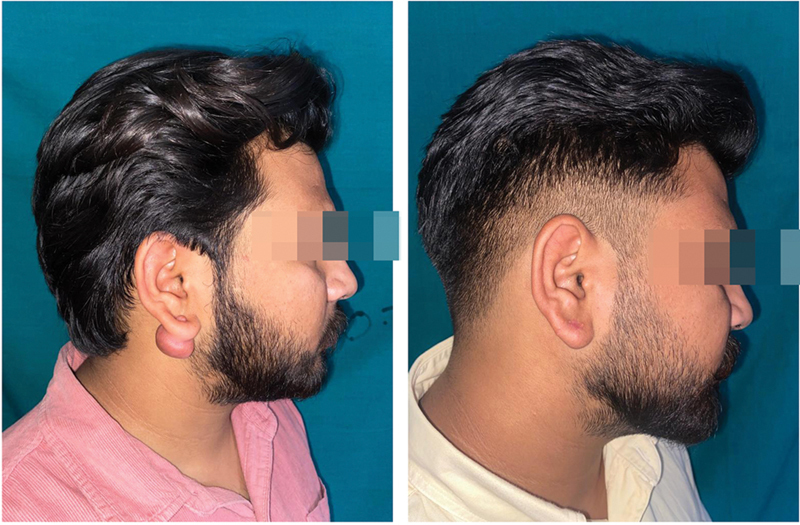
Patient A outcome before and after 1 year following surgical excision of right ear keloid followed by intramarginal combination therapy of 5-fluorouracil (5-FU) and triamcinolone acetonide (TCA) along with compression therapy.

Overall, pain was reported at the keloid site by 9 patients in group A and 7 in group B. Ulceration at the keloid site was seen in 2 patients in group A, and burning sensation was reported by 3 patients in group A and 1 in group B. Wound dehiscence was noted in 1 patient and transient hyperpigmentation in 2 patients from group A.

Based on the VSS, the outcome on follow-up had an average of 3.5 at 3 months, 4.2 at 6 months, and 4.8 at 1 year in group A, and 3.8 at 3 months, 4.7 at 6 months, and 5.4 at 1 year in group B ( Fig. 3 ).

## Discussion

Keloid formation can be induced by skin trauma in predisposed individuals, which can be secondary to body piercings, burns, lacerations, folliculitis, acne, or surgical wounds. They are seen more commonly in dark-skinned individuals with a slight predominance in female gender. Keloids have a familial predisposition and rarely regress. Fibroblasts in keloids overproduce type I procollagen besides lower rates of apoptosis along with elevated transforming growth factor-β.

Keloids are known to be onerous to manage attributable to their unknown pathophysiology, which is still debatable and under evaluation.

Keloids are notorious owing to their recurrence even after prolonged management. Multimodal approach is still under evaluation and can be opted to alleviate the morbidity of the patients while achieving recurrence-free results with minimal complications.

Combination therapy is evolving in the management of keloids. In the present study, TCA with/without 5-FU was used intramarginally after complete surgical excision of keloids in ears, followed by compression therapy.

Keloids of ears evaluated in our study were mostly incidental at the sites of previous ear piercings. Keloids were seen most commonly in the third decade of life, which was similar to the study of Berman et al, which reported that the keloids peak in 10 to 20 years of age. 10

Keloids are infamous for their recurrence. Recurrence rate of keloids with surgical excision alone can range from 80 to 100% as mentioned by Gold at al. In our study, 81.7% cases had no previous history of medical/surgical intervention in the management of keloids and these 98 patients had consulted for the first time. 11

Recurrence rate with TCA was 33% over 1 and 50% over 5 years with TCA in the study by Morelli Coppola et al, which is similar to our study with recurrence rate of 38.3% over 2 years with TCA alone and 21.7% with combination of TCA and 5-FU. 4

In 1999, Fitzpatrick reported the combination therapy of TCA with 5-FU in the management of keloids with pulsed dye therapy, reporting a 9-year experience administering > 5,000 injections to > 1,000 patients. He found that by mixing 1 mg/mL TCA with 5-FU (by adding 0.1 mL of 10 mg/mL TCA to 0.9 mL of 50 mg/mL 5-FU), efficacy was improved and injections were less painful. 12

Combination therapy of 5-FU and TCA can decrease the recurrence rate of keloids. Recurrence rate with 5-FU and TCA was reported to be around 25 to 47% in a study by Bijlard at al, which in our study was seen in 21.7% patients when combined with compression therapy. 13

Side effects are seen more in combination therapy of 5-FU and TCA than TCA alone, but are mild in severity and localized. No generalized complications were noted. Ulcerations healed gradually over time without any prolonged ailment. Pain was managed with oral anti-inflammatory drugs. Wound dehiscence was managed with aseptic dressings with collagen granules. Transient hyperpigmentation at the keloids site resolved over a span of 3 to 4 weeks without any intervention as also noted by Gupta and Kalra. 14 Side effects of 5-FU and TCA in our study are similar to the results of Shah et al. 15

Incorporating silicone gel sheets in compression therapy can further reduce the chances of recurrence of keloids as was also seen in the results obtained by Tahir et al. In their analysis of the management of keloids, combination treatment with compression devices and silicone gel sheets had the lowest recurrence rate when compared with compression device or silicone gel sheets alone. 5

VSS assessment of the outcomes was noted to be marginally higher in group B than A, which can be attributed to lower recurrence seen with combination therapy of 5-FU with TCA than TCA alone.

A larger sample size and further evaluation is required in understanding the pathophysiology of keloids in a better way, and in allocating the best treatment modality for keloids. Pitfalls in this study include the materials of the customizable instruments used for the compression therapy. Further assessment of different materials in these customizable instruments can be done for earning even better outcomes.

## Conclusion

Multimodal approach of combination therapy of intramarginal 5-FU and TCA with compression therapy after surgical excision of keloids in ears yields lower recurrence rate when compared with TCA alone. Chances of recurrence are more common in males than females. Though intramarginal 5-FU in combination with TCA has more localized side effects than TCA alone yet lower recurrence rate and better results in the long term can overcome the mild severity of these side effects.
